# The Number of Central Lymph Nodes on Preoperative Ultrasound Predicts Central Neck Lymph Node Metastasis in Papillary Thyroid Carcinoma: A Prospective Cohort Study

**DOI:** 10.1155/2020/2698659

**Published:** 2020-04-14

**Authors:** Luying Gao, Juanjuan Wang, Yuxin Jiang, Qiong Gao, Ying Wang, Xuehua Xi, Bo Zhang

**Affiliations:** ^1^Department of Ultrasound, Chinese Academy of Medical Sciences & Peking Union Medical College Hospital, Beijing, China; ^2^Department of Ultrasound, China-Japan Friendship Hospital, Beijing, China

## Abstract

To evaluate the effectiveness of the number of central compartment lymph nodes (CLNs) on ultrasound (US) in predicting CLN metastasis (CLNM). We prospectively studied 309 papillary thyroid cancer (PTC) patients who underwent thyroidectomy with CLN dissection at our center from May 2017 to July 2017. The number and features of CLNs were evaluated preoperatively *via* US. All US examinations were performed using a Philips iU 22 or a GE Logiq 9 machine. Correlations between CLNs observed *via* preoperative US and amount of CLNM were evaluated. We found that ≥2 CLNs on the preoperative US was associated with CLNM (*P* < 0.01). For this feature, the sensitivity, specificity, and area under the curve (AUC) were 54.3%, 66.1%, and 0.61, respectively. The presence of both suspected metastasis and ≥2 CLNs on US had a specificity of 86.5%. In addition, ≥3 CLNs on preoperative US was associated with large-volume CLNM (>5 metastatic CLNs) (*P* < 0.01). For this feature, the sensitivity, specificity and AUC were 54.8%, 74.5% and 0.65, respectively. The presence of both suspected metastasis and ≥3 CLNs on US had a specificity of 84.9%. The presence of suspected metastasis and/or ≥3 CLNs had a sensitivity of 80.6%. Our results suggest that ≥2 and ≥ 3 CLNs on preoperative US may serve as ancillary preoperative markers for predicting CLNM.

## 1. Introduction

Papillary thyroid cancer (PTC) accounts for 85% of the cases of differentiated thyroid cancer. Despite a generally benign course of PTC, early cervical lymph node (CLN) metastasis may occur. Twenty percent to 60% of PTC patients have LN involvement, with malignant LNs more frequently located in the central compartment [1–9]. PTC patients with LN involvement have a poorer prognosis than those without LN involvement [10]. Large-volume LN metastasis (LNM) is defined as the presence of more than five metastatic LNs and has been shown to be associated with a 20% risk of local recurrence but also an increased risk of distant metastasis [11, 12].

Ultrasonography is the preferred screening modality for preoperative assessment of regional LNs in PTC patients [13]. However, the deep location of central compartment LNs (CLNs) renders it difficult to delineate these LNs on ultrasound (US). Preoperative neck US has a low sensitivity (44.4%) for detecting CLN metastasis (CLNM) [14]. A recent trend toward routine central LN dissection avoiding radioactive treatment is still debated [15–17], and prophylactic central neck dissection (pCND) in patients with clinically uninvolved cN0 has been ineffective in some studies and is accompanied by postoperative complications [18, 19]. Thus, finding preoperative features to predict CLNM is crucial, particularly large-volume CLNM, for PTC patients. This prospective study sought to assess the value of the number of CLNs on US for predicting CLNM and large-volume CLNM in patients with PTC.

## 2. Patients and Methods

### 2.1. Patients and Definitions

**Figure 1:**
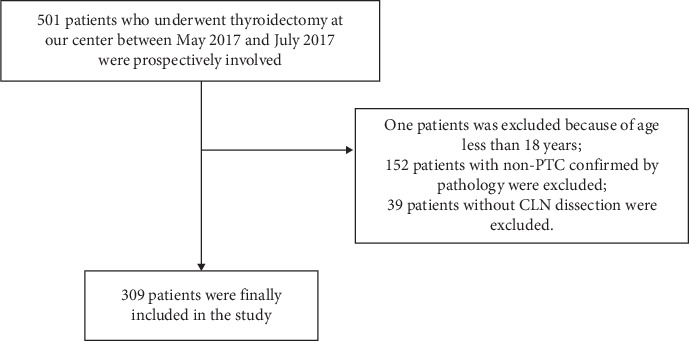
The flow chart of selection of papillary thyroid carcinoma patients.

We prospectively studied 501 adult (>18 years) patients with pathologically proven PTC who underwent thyroidectomy at our center between May 2017 and July 2017. Two experienced radiologists at our center evaluated preoperative US for CLNs. We included patients who underwent total or nearly total thyroidectomy with CLN dissection (*n* = 309) (Figure 1).

Hashimoto's thyroiditis was determined pathologically. Extracapsular invasion included both microscopic and gross invasion by the tumour. Based on the number of metastatic central LNs, patients were divided into three groups according to CLNM status: no CLNM, small-volume CLNM (≤5 metastatic CLNs), or large-volume LNM (>5 metastatic CLNs) [11, 12].

### 2.2. Thyroid US Examination

All US examinations were performed using a Philips iU 22 machine (Philips Healthcare, Eindhoven, Netherlands) or a GE Logiq 9 machine (GE Healthcare, Milwaukee, WI, USA); all US machines were equipped with a 5–12 MHz linear-array transducer. The ultrasonographic examinations included central cervical LNs and the size of the thyroid nodules. In cases involving multifocal PTC, the largest thyroid nodule on US was recorded. The number, size, shape, and structure of LNs, cystic appearance, hyperechogenic punctuations, hyperechogenic hilum, and vascularity, were evaluated by US. Suspicious sonographic features of central cervical LNs included enlargement (short axis >5 mm), loss of the fatty hilum, a rounded rather than oval shape, hyperechogenicity, cystic change, calcifications, and peripheral vascularity. LNs were considered suspicious when 1 or more of the suspicious US findings were present [1].

US images were prospectively evaluated by two radiologists who were experienced in thyroid US and were blinded to patient clinical data and pathological results. Discrepancy between the two assessments was resolved by consensus after discussion.

The study protocol was approved by the Ethics Committee of our hospital.

### 2.3. Postoperative Complications

Major postoperative complications were recorded including neck hematoma requiring surveillance, transient or permanent unilateral recurrent nerve injury, and transient or permanent hypoparathyroidism. Serum calcium levels were assayed based on the evolution of clinical and biochemical parameters. In the case of hypocalcaemia, parathyroid hormone (PTH) levels were determined. Hypoparathyroidism was considered permanent if it lasted more than 6 months and required medical therapy with normal serum PTH levels. Paralysis of the recurrent laryngeal nerve was confirmed by laryngoscopy and considered permanent if it persisted for more than 6 months.

### 2.4. Statistical Analysis

Quantitative data were presented as means ± standard deviations (SDs). Qualitative data were presented as frequencies. A Shapiro–Wilk test was used to evaluate whether data were normally distributed. Between-group differences for nonparametric data were analysed using a Mann–Whitney *U* test. Parametric data were analysed using an unpaired *t*-test to evaluate between-group differences. A chi-square test with Yates' correction and Fisher's exact test were used to compare categorical variables. Sensitivities, specificities, positive predictive values (PPVs), negative predictive values (NPVs), and accuracies were calculated by comparing pathological findings. Receiver operating characteristic (ROC) curve analyses were used to calculate optimal cut-off values. A logistic regression model was used to evaluate risk factors. *P* < 0.05 was considered statistically significant. Statistical analyses were performed using SPSS software (version 19.0, SPSS Inc., Chicago, IL, USA).

## 3. Results

### 3.1. Demographic and Baseline Features of the Patients

The demographic and baseline features of the patients are listed in Table 1. Among the 309 patients, 171 (55.3%) patients had no CLNM, 107 (34.6%) patients had small-volume CLNM, and 31 (10.0%) patients had large-volume CLNM. The mean age of the patients was 43.6 ± 10.6 years. The majority of the patients were female (*n* = 234; 75.7%). The mean size of the primary tumour was 1.0 ± 0.6 cm, and 113 (36.2%) cases involved a tumour >1 cm. We examined all case records and identified the following postoperative complications: hematoma in surgical bed in 13 cases (4.2%), permanent recurrent laryngeal nerve injury in 2 cases (0.6%; laryngoscopy showing paralysis persisting beyond 6 months), and permanent hypoparathyroidism requiring replacement therapy in 2 cases (0.6%).

### 3.2. Clinical and US Features and Status of Central Lymph Node Metastases

We analysed the correlation between features and CLNM. The younger group of patients (age <55 years old) tended to have a high prevalence of CLNM compared to older patients (*P* < 0.01). Relative to a smaller tumour, a larger tumour (>1.0 cm) was significantly associated with CLNM (*P* < 0.01). The presence of suspected metastatic CLNs on preoperative US was significantly associated with CLNM (*P* < 0.01). Extracapsular invasion was also significantly associated with CLNM (*P* < 0.01). We further analysed the correlation between features and the number of CLNMs. Larger tumour size (>1.0 cm) was significantly associated with large-volume CLNM (*P* < 0.01). The presence of suspected metastatic CLNs on preoperative US was significantly associated with large-volume CLNM (*P* < 0.01) (Table 1).

Table 2 shows the sensitivity, specificity, positive predictive value (PPV), negative predictive value (NPV), accuracy, area under the curve (AUC) of younger age (<55 years), larger tumour size (>1.0 cm), suspected metastatic CLNs on preoperative US, and extracapsular invasion for CLNM.  Table 3 shows the sensitivity, specificity, positive predictive value (PPV), negative predictive value (NPV), accuracy, and area under the curve (AUC) of larger tumour size (>1.0 cm) and suspected metastatic CLNs on preoperative US for large-volume CLNM.

### 3.3. Risk Factors for CLNM and Large-Volume CLNM

To identify independent factors associated with CLNM and large-volume CLNM, significant variables were entered into the final models for multivariate testing. Suspected metastatic CLN on preoperative US was an independent risk factor for CLNM (odds ratio [OR] = 1.64 (95%confidence interval (95% CI) 1.23–2.19), *P* < 0.01), and suspected metastatic CLN on preoperative US was an independent risk factor for large-volume CLNM (OR = 1.96 (95% CI 1.25–3.05), *P*=0.003). Tumour size (>1 cm) was an independent risk factor for CLNM (OR = 2.24 (95% CI 1.33–3.77), *P*=0.002). Younger age (<55 years) was another independent risk factor for CLNM (OR = 8.00 (95% CI 3.13–20.41), *P* < 0.01).

Table 4 displays the sensitivity and specificity of each suspected metastatic US signs (enlargement, loss of the fatty hilum, a rounded rather than oval shape, hyperechogenicity, cystic change, calcifications, and peripheral vascularity) for CLNM.

### 3.4. The Number of CLNs on Preoperative US and Status of Central Lymph Node Metastases

The number of CLNs on preoperative US was significantly associated with CLNM (*P* < 0.01). ROC curves demonstrated that 2 was the best cut-off value for CLNs on preoperative US. The sensitivity, specificity, PPV, NPV, accuracy, and AUC for ≥2 visible CLNs were 54.3%, 66.1%, 56.4%, 64.2%, 60.8%, and 0.61 (95% CI 0.55–0.67), respectively. We further assessed the added diagnostic value provided by the criteria of ≥2 CLNs with respect to suspected metastatic CLNs. The presence of both suspected metastasis and ≥2 CLNs on US was significantly associated with CLNM (*P* < 0.01). The sensitivity, specificity, PPV, NPV, accuracy, and AUC were 40.6%, 86.5%, 70.9%, 64.3%, 66.0%, and 0.64 (95% CI, 0.57–0.70), respectively. The presence of suspected metastasis and/or ≥2 CLNs was also significantly associated with CLNM (*P* < 0.01). The sensitivity, specificity, PPV, NPV, accuracy, and AUC were 63.0%, 55.6%, 53.4%, 65.1%, 58.9%, and 0.59 (95% CI, 0.53–0.66), respectively (Table 2).

The number of CLNs on preoperative US was significantly associated with large-volume CLNM (*P* < 0.01). ROC curves demonstrated that 3 was the best cut-off value for CLNs on preoperative US. The sensitivity, specificity, PPV, NPV, accuracy, and AUC for ≥3 CLNs were 54.8%, 74.5%, 19.3%, 93.9%, 72.4%, and 0.65 (95% CI, 0.54–0.75), respectively. We further assessed the added diagnostic value provided by the criteria of ≥3 CLNs with respect to suspected metastatic CLNs. The presence of both suspected metastasis and ≥3 CLNs on US was significantly associated with large-volume CLNM (*P* < 0.01). The sensitivity, specificity, PPV, NPV, accuracy, and AUC were 45.2%, 84.9%, 25.0%, 93.3%, 80.9%, and 0.65 (95% CI, 0.54–0.76), respectively. The presence of suspected metastasis and/or ≥3 CLNs was also significantly associated with CLNM (*P* < 0.01). The sensitivity, specificity, PPV, NPV, accuracy, and AUC were 80.6%, 58.3%, 17.7%, 96.4%, 60.5%, and 0.70 (95% CI, 0.60–0.79), respectively (Table 3).

### 3.5. The Diagnostic Value of CLN Number for PTC Patients with Hashimoto's Thyroiditis

We calculated the diagnostic value of the number of CLNs for those PTC patients combined with Hashimoto's thyroiditis (*N* = 63). ≥2 CLNs on preoperative US was significantly associated with CLNM (*P*=0.02). The sensitivity, specificity, accuracy, and AUC for ≥2 CLNs were 81.8%, 43.3%, 63.5%, and 0.63 (95% CI, 0.49–0.77), respectively.

We calculated the diagnostic value of the number of CLNs for those PTC patients combined without Hashimoto's thyroiditis (*N* = 246). ≥2 CLNs on preoperative US was significantly associated with CLNM (*P* < 0.01). The sensitivity, specificity, accuracy, and AUC for ≥2 CLNs were 45.9%, 71.5%, 60.2% and 0.59 (95% CI, 0.52–0.67), respectively. ≥3 CLNs on preoperative US was significantly associated with large-volume CLNM (*P* < 0.01). The sensitivity, specificity, accuracy, and AUC for ≥3 CLNs were 50.0%, 81.7%, 78.0%, and 0.67 (95% CI, 0.55–0.79), respectively.

## 4. Discussion

In our investigation, younger age, larger tumour size, extracapsular invasion, and suspected metastatic CLNs on preoperative US were associated with CLNM of PTC patients. Furthermore, we found that ≥2 CLNs was associated with CLNM, and ≥3 CLNs was associated with large-volume CLNM. For predicting CLNM, the presence of suspected metastasis and/or ≥2 CLNs had a relative high sensitivity. For predicting large-volume CLNM, the presence of both suspected metastasis and ≥3 CLNs had a relative high specificity, and the presence of suspected metastasis and/or ≥3 CLNs had a relative high sensitivity. In our study, Hashimoto's thyroiditis was not associated with CLNM, and for patients with Hashimoto's thyroiditis, the diagnostic value of the number of CLNs is still positive.

Due to the location of CLNs, it is difficult to identify their features *via* US. As a result, preoperative US exhibits low sensitivity for detecting CLNM [14]. In our study, 49.3% of the PTC patients with CLNM had suspected metastatic CLNs on preoperative US; 20.2% of the PTC patients with suspected metastatic CLNs had large-volume CLNM based on pathological findings. These results show that the accuracy of preoperative US for evaluating CLNs is unreliable. Since our study is a prospective study, the number and US feature of LNs are of great concern when examining the CLNs. Our findings demonstrate that ≥2 CLNs and ≥3 CLNs are valuable preoperative clinical parameters for predicting CLNM or large-volume CLNM. Moreover, our findings suggest that the number of CLNs on US provides added diagnostic value to that of suspected metastasis observed in CLNs *via* US. The high specificity and PPV afforded by presentation of both suspected metastasis and ≥2 CLNs could help rule in CLNM if the outcome is positive. The high sensitivity and NPV afforded by presentation of suspected metastasis and/or ≥3 CLNs may help rule out large-volume CLNM if the outcome is negative. These findings suggest that treatment strategies should be modified for patients with a relatively large number of CLNs and suspected metastatic CLNs on preoperative US. Since large-volume pathologic lymph node metastasis is an important recurrence factor by ATA risk stratification system [1] and more than half of the patients operated on showed no CLNM, ≥3 CLNs on preoperative US may serve as a cut-off marker for prophylactic central node dissection of patients with PTC. Moreover, the results showed that ≥2 or ≥3 CLNs on preoperative US were not an independent risk factor for predicting CLNM and may serve as an ancillary marker with respect to suspected metastatic CLNs.

Age at diagnosis of thyroid cancer is identified as an independent predictor of disease-specific survival in most published staging systems [20, 21]. A previous study showed that large-volume CLNM was more frequently observed in younger patients [19]. This phenomenon was also demonstrated by a recent international multicentre retrospective study in which the age cut-off was shifted from 45 to 55 years [22]. Our results are comparable to these previously reported findings. Younger age is a valuable parameter for detecting CLNM.

Tumour size was significantly associated with LN metastasis in patients with PTC. Kim et al. found that compared with patients with smaller tumours, patients with larger tumours had a higher rate of postoperative recurrence, invasion, and CLN metastasis [23, 24]. Our results, which are comparable to those reported in previous studies, showed that larger tumour size (≥1 cm) was correlated with CLNM and large-volume CLNM. Our findings suggest that age and tumour size may serve as preoperative ancillary markers for determining the extent of surgery.

Hashimoto's thyroiditis patients often have central lymph node enlargement [24]. In our study, Hashimoto's thyroiditis was not associated with CLNM. Moreover, we calculated the diagnostic value of the number of CLNs for those PTC patients combined with Hashimoto's thyroiditis. ≥2 CLNs on preoperative US was significantly associated with CLNM for those PTC patients combined with Hashimoto's thyroiditis. For predicting CLNM, the sensitivity, specificity, accuracy, and AUC for ≥2 CLNs were 81.8%, 43.3%, 63.5%, and 0.63, respectively. It seems that in PTC patients with Hashimoto's thyroiditis, the diagnostic value of the number of CLNs declines, but is still meaningful.

The 2015 ATA guideline suggests active surveillance for nodules <1 cm in the absence of evidence of extrathyroidal extension, metastatic cervical lymph nodes, or distant metastases [1]. However, the treatment of micropapillary thyroid cancer is still controversial. In clinical practice, still a large number of nodules <1 cm received FNA or surgical resection. There are several reasons for these patients with thyroid malignant nodules <1 cm to select FNA and surgery. In China, patients have poor compliance with active follow-up. During the follow-up, patients need to receive imaging examinations, blood tests, etc., but the medical insurance system does not cover the examinations in the outpatient clinic. Due to time costs, labor costs, and other expense issues, doctors and patients in China may be more inclined to choose surgery. Another important reason is more than 30% cervical lymph node metastasis in CN0 PTC and extrathyroidal extension [25, 26]. With the application of ACR-TIRADS and ATA guidelines in recent years, this situation has been improving [27].

Our study has several limitations. First, all the patients underwent thyroidectomy, which may have led to selection bias resulting in underestimation of NPV and overestimation of PPV for preoperative US, younger age and larger tumour size. Second, our study included 501 patients, only 31 of whom had large-volume CLNM. Future studies that involve more subjects may produce more accurate results.

## 5. Conclusion

The criteria of ≥2 CLNs and ≥3 CLNs on preoperative US have effective diagnostic value for predicting CLNM and large-volume CLNM in patients with PTC. The number of CLNs on preoperative US may provide additional information for predicting CLNM. In PTC patients with Hashimoto's thyroiditis, the diagnostic value of the number of CLNs decreases, but is still valuable. Our results suggest that treatment may be modified for PTC patients with a relatively large number of CLNs and suspected metastatic CLNs on preoperative US.

## Figures and Tables

**Table 1:** The demographic and baseline features and status of central lymph node metastases in 309 patients with PTC.

	No CLNM	Small-volume CLNM	Large-volume CLNM	*P*
Age				<0.01
<55	131 (49.8%)	103 (39.2%)	29 (11.0%)	
≥55	40 (88.9%)	4 (8.9%)	2 (4.4%)	

Sex				0.29
Male	36 (48.0%)	29 (38.7%)	10 (13.3%)	
Female	135 (57.7%)	78 (33.3%)	21 (8.9%)	

Size of primary tumour				<0.01
≤1 cm	123 (62.8%)	60 (30.6%)	13 (6.6%)	
>1 cm	48 (42.5%)	47 (41.6%)	18 (15.9%)	

Hashimoto's thyroiditis				0.72
Yes	44 (54.3%)	27 (33.3%)	10 (12.3%)	
No	127 (55.7%)	80 (35.1%)	21 (9.2%)	

Presence of CLNs on US				0.05
Yes	111 (52.6%)	73 (34.6%)	27 (12.8%)	
No	60 (60.6%)	34 (34.3%)	4 (4.0%)	

Suspected metastatic CLNs on US				<0.01
Yes	41 (37.6%)	46 (42.2%)	22 (20.2%)	
No	130 (65.0%)	61 (30.5%)	9 (4.5%)	

CLNM, central compartment lymph node metastasi. CLN, central compartment lymph nodes. US, ultrasound.

**Table 2:** Diagnostic efficiency of features for CLNM status.

	Sensitivity (%)	Specificity (%)	PPV (%)	NPV (%)	Accuracy (%)	AUC
Age <55 years	95.7	23.4	50.2	87.0	55.7	0.59
Tumour size >1.0 cm	47.1	71.9	57.5	62.8	60.8	0.60
≥2 CLNs	54.3	66.1	56.4	64.2	60.8	0.61
Suspected metastatic CLNs	49.3	76.0	62.4	65.0	64.1	0.63
Suspected metastasis and ≥2 CLNs	40.6	86.5	70.9	64.3	66.0	0.64
Suspected metastasis and/or ≥2 CLNs	63.0	55.6	53.4	65.1	58.9	0.59

CLNM, central compartment lymph node metastasis. CLN, central compartment lymph nodes. AUC, area under the curve. PPV, positive predictive value. NPV, negative predictive value.

**Table 3:** Diagnostic efficiency of features for large-volume CLNM.

	Sensitivity (%)	Specificity (%)	PPV (%)	NPV (%)	Accuracy (%)	AUC
Tumour size >1.0 cm	58.1	65.8	15.9	93.4	65.0	0.62
≥3 CLNs	54.8	74.5	19.3	93.7	72.4	0.65
Suspected metastatic CLNs on preoperative US	71.0	68.7	20.2	95.5	68.9	0.70
Suspected metastasis and ≥3 CLNs	45.2	84.9	25.0	93.3	80.9	0.65
Suspected metastasis and/or ≥3 CLNs	80.6	58.3	17.7	96.4	60.5	0.70

CLNM, central compartment lymph node metastasis. CLN, central compartment lymph nodes. AUC, area under the curve. PPV, positive predictive value. NPV, negative predictive value.

**Table 4:** The diagnostic efficiency of suspected metastatic ultrasound signs.

Signs	Sensitivity, (%)	Specificity, (%)	No CLNM, (%)	CLNM, (%)
Enlargement	16.2	95.2	8 (25.8)	23 (74.2)
Loss of the fatty hilum	45.8	79.0	35 (35.0)	65 (65.0)
Round shape	16.2	91.0	15 (49.5)	23 (60.5)
Microcalcifications or hyperechogenicity	12.0	97.0	5 (22.7)	17 (77.3)
Cystic aspect	11.3	100.0	0 (0.0)	16 (100.0)
Peripheral vascularity	14.8	98.2	3 (12.5)	21 (87.5)

CLNM, central compartment lymph node metastasis.

## Data Availability

The data used to support the findings of this study are available from the corresponding author upon request.
